# Plant organelle RNA editing and its specificity factors: enhancements of analyses and new database features in PREPACT 3.0

**DOI:** 10.1186/s12859-018-2244-9

**Published:** 2018-07-03

**Authors:** Henning Lenz, Anke Hein, Volker Knoop

**Affiliations:** 1 IZMB – Institut für Zelluläre und Molekulare Botanik, Abteilung Molekulare Evolution, Universität Bonn, Kirschallee 1, 53115 Bonn, Germany; 2 IBG-2: Plant Sciences, Forschungszentrum Jülich GmbH, 52425 Jülich, Germany

**Keywords:** Pentatricopeptide repeat (PPR) proteins, Pyrimidine exchange RNA editing, Mitochondria, Chloroplasts, RNA-binding proteins

## Abstract

**Background:**

Gene expression in plant chloroplasts and mitochondria is affected by RNA editing. Numerous C-to-U conversions, accompanied by reverse U-to-C exchanges in some plant clades, alter the genetic information encoded in the organelle genomes. Predicting and analyzing RNA editing, which ranges from only few sites in some species to thousands in other taxa, is bioinformatically demanding.

**Results:**

Here, we present major enhancements and extensions of PREPACT, a WWW-based service for analysing, predicting and cataloguing plant-type RNA editing. New features in PREPACT’s core include direct GenBank accession query input and options to restrict searches to candidate U-to-C editing or to sites where editing has been documented previously in the references. The reference database has been extended by 20 new organelle editomes. PREPACT 3.0 features new modules “EdiFacts” and “TargetScan”. EdiFacts integrates information on pentatricopeptide repeat (PPR) proteins characterized as site-specific RNA editing factors. PREPACT’s editome references connect into EdiFacts, linking editing events to specific co-factors where known. TargetScan allows position-weighted querying for sequence motifs in the organelle references, optionally restricted to coding regions or sequences around editing sites, or in queries uploaded by the user. TargetScan is mainly intended to evaluate and further refine the proposed PPR-RNA recognition code but may be handy for other tasks as well. We present an analysis for the immediate sequence environment of more than 15,000 documented editing sites finding strong and different bias in the editome data sets.

**Conclusions:**

We exemplarily present the novel features of PREPACT 3.0 aimed to enhance the analyses of plant-type RNA editing, including its new modules EdiFacts integrating information on characterized editing factors and TargetScan aimed to analyse RNA editing site recognition specificities.

**Electronic supplementary material:**

The online version of this article (10.1186/s12859-018-2244-9) contains supplementary material, which is available to authorized users.

## Background

Nearly 30 years after the discovery of C-to-U RNA editing in plant mitochondria [1–3] and quickly thereafter also in chloroplasts [4], the field has recently expanded tremendously in several directions of research [5–7]. After the initial characterization of a first chloroplast [8] and a first mitochondrial RNA editing factor [9] numerous such proteins continue to be characterized, quickly outdating published compilations [5, 10–12] by ever more new additions [13–15]. The key factors in RNA editing site recognition are pentatricopeptide repeat (PPR) proteins, which are encoded by tremendously enlarged gene families with hundreds of members in plants [16–19].

The arrays of PPRs are key to specifically recognizing the RNA sequences upstream of cytidines targeted for conversion into uridines via deamination. PPR proteins serving as editing factors have a unique makeup of alternating P-, L- and S-type PPRs with distinct amino acid conservation profiles. Moreover, PPR proteins characterized as editing factors carry carboxyterminal protein domain additions, minimally “E” (extension) domains, frequently followed by the so-called “DYW” domain. The latter in particular is of fundamental interest owing to its significant similarity to cytidine deaminases, which likely provides the biochemical activity for C-to-U conversion [20–23].

Intriguingly, DYW-type PPR proteins that were previously believed to be plant-specific, have recently been identified in very distant evolutionary lineages of eukaryotes where their presence likewise seems to be connected to mitochondrial RNA editing of the C-to-U type [24–27].

A PPR-RNA recognition code has been proposed [28], which is currently subject to further amendments and experimental testing in vivo and in vitro [29–32]. Linking RNA editing events or other transcript targets to specific PPR array sequences and vice versa is becoming an exciting field for bioinformatic approaches and for potential future applications using artificially designed PPR arrays [31, 33, 34]. The former issue becomes obvious, for example, when numbers of editing events both in mitochondria and in chloroplasts literally run into thousands, such as in the lycophytes [35–37].

The PREPACT WWW service developed in our group [38, 39] aimed for (i) standardizing RNA editing annotation and nomenclature, (ii) making the vast and ever-increasing amount of editing information available with manually curated reference editomes (i.e. the sets of editing sites determined with extensive cDNA analysis for organelle genomes), and (iii) helping to analyze and predict RNA editing in organelle sequence data. We here demonstrate an update of PREPACT in version 3.0 with respect to its “classic” features, but now also aiming to address the interplay between RNA editing sites and their cognate PPR-type specificity factors. Information on the latter are now included in a novel database module “EdiFacts” and the possibility to experimentally scan for potential RNA targets is realized with the new “TargetScan” module. We present the new features of PREPACT’s core functionalities, 20 new editome reference addendums, demonstrate the functionalities of EdiFacts and TargetScan and discuss future issues and developments of RNA editing analysis, especially those related to PPR-RNA recognition.

## Results

### PREPACT editome reference extensions

**Fig. 1:**
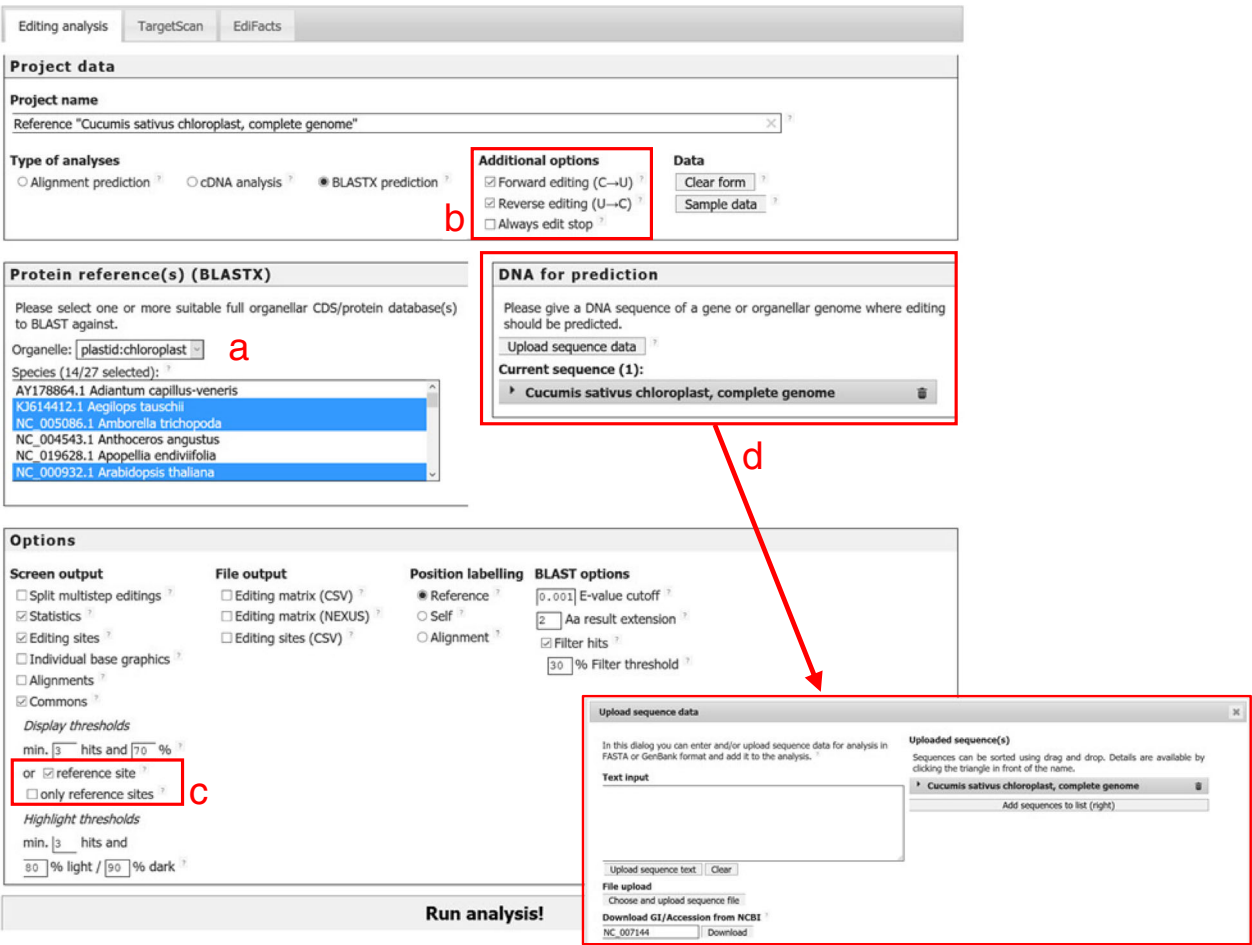
The updated PREPACT 3.0 (www.prepact.de) input interface offering several major enhancements. Several new organelle reference editomes have been added as described in the text. Here exemplarily shown is the selection of the 14 angiosperms out of altogether 27 chloroplast editomes now available (**a**). U-to-C editing and C-to-U editing may now be selected individually, and a new feature allows prognosis of U-to-C reverse editing to remove stop codons even when no conserved arginine or glutamine codons would be restored (**b**). Editing prediction may be restricted to sites where RNA editing has been identified previously in at least one of the references or to always include such sites in the output (**c**) even when below the overall commons thresholds defined above. As an alternative to FASTA-formatted input of a query, GenBank database accessions may simply be given as exemplarily shown for the *Cucumis sativus* cpDNA (**d**). Overall enhanced handling of the sequence input is particularly relevant for the multiple sequence alignment analysis modes (Additional file 1)

One core component of PREPACT’s functionality is a set of mitochondrial and chloroplast genomes with curated and standardized RNA editing site information [39]. A user-defined selection of these reference editomes can be used to simultaneously identify organelle protein-coding genes and candidate RNA editing sites in an unannotated organelle nucleotide sequence query using PREPACT’s BLASTX mode (Fig. 1).

We have added several new organelle editome references with reliably determined editing site identifications (Table 1). The 14 chloroplast editomes of the flowering plants *Amborella trichopoda* [40], *Aegilops tauschii* [41], coconut *Cocos nucifera* [42], cucumber *Cucumis sativus* [40, 43], cotton *Gossypium hirsutum* [44], the orchid *Phalaenopsis aphrodite* [45], the duckweed *Spirodela polyrhiza* [46] and the mung bean *Vigna radiata* [47], the gymnosperm *Ginkgo biloba* [48], the liverwort *Apopellia endiviifolia* [49], the lycophyte *Selaginella uncinata* [36], the horsetail *Equisetum hyemale* [50] and the ferns *Ophioglossum californicum* and *Psilotum nudum* [51] have been added to the plastome references previously included in PREPACT 2.0 [39]. The gymnosperm *Ginkgo*, the early-branching angiosperm *Amborella* and the jungermanniid liverwort *Apopellia* fill important taxonomic gaps. Similarly, the horsetail *Equisetum* and the club moss *Selaginella* are important addendums as taxa representing the full range of a taxon lacking chloroplast editing altogether [50] and the most heavily edited organelle transcriptome known so far with more than 3400 sites of C-to-U editing [36].

**Table 1:** New organelle editome entries added to the PREPACT reference library

Organelle Species	GenBank accession	RNA editing study	Editing sites
14 new chloroplast editomes			
*Aegilops tauschii*	KJ614412	[41]	49
*Amborella trichopoda*	NC_005086	[40]	156
*Apopellia endiviifolia*	NC_019628	[49]	54
*Cocos nucifera*	NC_022417	[42]	98
*Cucumis sativus*	NC_007144	[43]	65
*Equisetum hyemale*	NC_020146	[50]	0
*Ginkgo biloba*	KP099648	[48]	263
*Gossypium hirsutum*	NC_007944	[44]	69
*Ophioglossum californicum*	NC_020147	[51]	232
*Phalaenopsis aphrodite*	NC_007499	[45]	47
*Psilotum nudum*	KC117179	[51]	30
*Selaginella uncinata*	AB197035	[36]	3488
*Spirodela polyrhiza*	NC_015891	[46]	74
*Vigna radiata*	AP014691	[47]	51
6 new mitochondrial editomes			
*Acrasis kona*	NC_026286	[26]	2
*Amborella trichopoda*	KF754799 KF754800 KF754801 KF754802 KF754803	[54]	824
*Cocos nucifera*	NC_031696	[55]	794
*Liriodendron tulipifera*	KC821969	[53]	827
*Ophioglossum californicum*	NC_030900	[52]	861
*Psilotum nudum*	KX171638 KX171639	[52]	731

NCBI-curated accessions (NC_) have been used preferentially when RNA editing information was retained. Numbers of editing sites (last column) indicate “applied” events in the RNA editing annotation of the PREPACT references. Numbers are occasionally higher than in the respective studies since duplicate annotations had to be used where multiple identical gene copies exist, mainly for those located in the chloroplast IR regions. The *Amborella trichopoda* and *Psilotum nudum* mitochondrial editome references were assembled from the separate mt chromosome sequence entries in these species. The resulting number of editome references now available in PREPACT 3.0 totals 52 (27 chloroplast and 25 mitochondrial entries)

Among the mitochondrial references, *Ophioglossum californicum* and *Psilotum nudum* are particularly valuable additions as the first fern mitochondrial editomes [52]. The editomes of *Liriodendron tulipifera* [53], *Amborella trichopoda* [54] and *Cocos nucifera* [55] are interesting additions representing early diverging angiosperm and monocot lineages. Moreover, we have added the mitochondrial DNA of the protist *Acrasis kona* where two events of plant-type C-to-U editing have recently been identified [26] as a further mitochondrial editome reference.

In some cases we refrained from adding further reference data owing to an evident lack of documented editing sites in the editomes at this stage like in the case of the rubber tree *Hevea brasiliensis* mitochondria [56], the soy bean *Glycine max* [57] and the *Utricularia reniformis* chloroplast [58] as well as the *Saccharum officinarum* [59] and *Cycas* organelle editomes [60, 61]. Likewise, we avoided editome data, which obviously seem to be affected by artefacts including non-canonical types of editing, which we could not reproduce in independent cDNA analyses, like in the chloroplast transcriptome studies of *Ipomoea batata* [62], *Deschampsia antarctica* [63] or *Elaeis guineensis* [64]. Altogether, the editome references now available in the updated PREPACT 3.0 database comprise 27 chloroplast and 25 mitochondrial entries.

### PREPACT input enhancements

The enhanced query interface of PREPACT 3.0 has several new options (Fig. 1). Searches for reverse U-to-C editing, previously only implemented as an optional addition, are now offered as an individual option allowing to restrict searches to U-to-C editing sites exclusively. Moreover, users may choose to restrict searches for candidate editing sites to positions where RNA editing at an orthologous position has previously been identified in at least one of the chosen references. A further option allows to always include such sites in the commons output even when below the overall threshold settings. The sequence input has been redesigned for dynamic handling of queries, now also allowing to simply enter database accession numbers, which are directly retrieved from GenBank/NCBI. Certainly, uploading or copy-pasting of FASTA-formatted data remains possible, too. Sequences are now checked on-the-fly to report formatting errors and to allow for immediate re-upload of corrected data. For the “Alignment” and “cDNA” analysis modes of PREPACT, multiple uploaded sequences can be sorted, deleted and rearranged between query and reference side using drag-and-drop, as we will detail below (see Additional file 1).

### PREPACT output enhancements

**Fig. 2:**
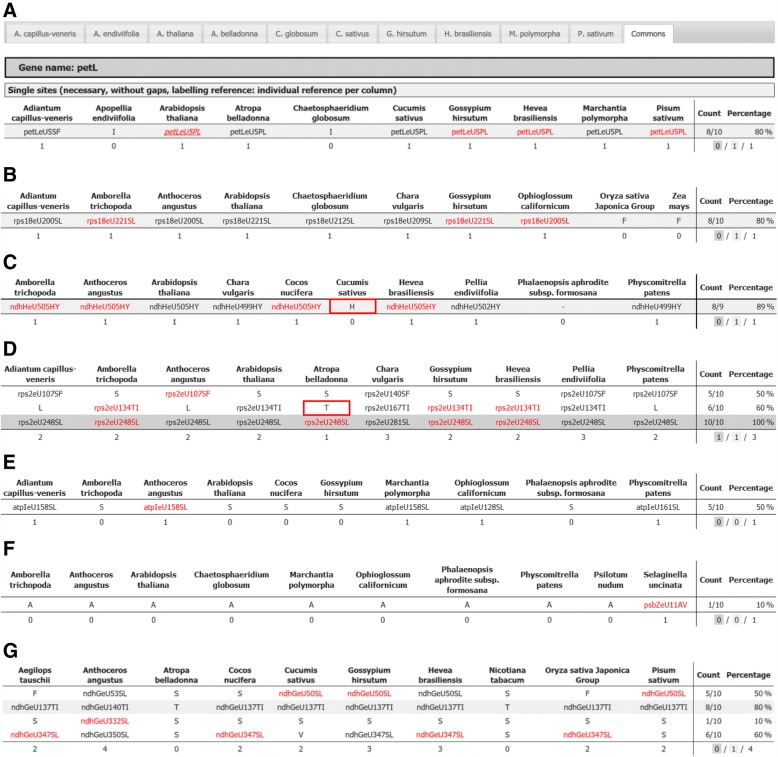
**a-g** Examples of the PREPACT 3.0 “commons” tab output for selected chloroplast queries as discussed in the text. For clarity of display, 10 of the now available 27 chloroplast references have been selected arbitrarily in each case. RNA editing prognoses are given in black when based on a “pre-edited” codon already present, but in red when based on a known RNA editing event in the respective organelle genome reference. The enhanced commons output now also displays amino acid identities for those references, which do not contribute to predict RNA editing events either because the unedited state is retained or because an inconvertible codon identity is present. The case of *petL* (**a**) and *rps18* (**b**) are given as examples discussed in the text. The use of hyphens is now restricted to cases of lacking homology, such as the case of the *ndh* genes in *Phalaenopsis* (**c**). Documentation of RNA editing event ndhHeU505HY in *Anthoceros* and *Hevea* (**c**) supported that it was previously overlooked in *Cucumis.* Like the case of rps2eU134TI in *Atropa* (**d**), these candidate editing sites (red boxes) are now confirmed as previously overlooked RNA editing events (Additional file 2). The cases of evolutionary ancestral editing events rps2eU107SF (**d**) and atpIeU158SL (**e**) in the hornwort *Anthoceros* lacking in angiosperms suggest a shift of amino acid conservation making RNA editing obsolete. Rarely, yet other cases may reflect isolated “orphan” editing such as in *Selaginella psbZ* (**f**) or RNA editing that merely serves to alter overall hydrophobicity than affecting relevant individual codons like in *ndhG* (**g**)

Examples for the PREPACT “commons” table output summarizing the RNA editing events predicted from comparisons to the selected references are shown in Fig. 2. PREPACT applies our previously suggested RNA editing site nomenclature, which is composed of the affected gene followed by an ‘e’ for editing, the nucleotide introduced by editing (C or U), the nucleotide position in the coding sequence and the resulting codon identity (if applicable) before and after editing to label editing sites [65]. The BLASTX output comprises the editing predictions from the selected references as individual tabs and a summary prediction as the final commons tab (Fig. 2a). The commons tab output now also displays amino acid identities in references that do not contribute to editing site prognoses either because of retention of the unedited state or due to an inconvertible codon at the corresponding position. This new feature helps significantly in the interpretation of the output because it immediately shows the variability of amino acids present for a candidate site predicted by only some of the references. For example, in the case of predicting editing event petLeU5PL converting a proline into a leucine (Fig. 2a), a chemically similar isoleucine (I) is present in the alga *Chaetosphaeridium* and in the liverwort *Apopellia*, which can be taken to further corroborate the likelihood of editing in the query (here *Wollemia nobilis* KP259800). Similarly, in the case of a leucine codon in *rps18* that is widely conserved in plants and algae and which requires editing from a serine codon in several references, a phenylalanine codon (F) is present in the Poales *Oryza* and *Zea* (Fig. 2b). Removal of the polar amino acid serine may be more important than the presence of either an aliphatic leucine or an aromatic phenylalanine at this position in the protein. A hyphen in the commons output is now restricted to cases where homology is lacking in a reference, e. g. in the case of the *ndh* genes lost altogether in the orchid *Phalaenopsis aphrodite* plastome (Fig. 2c).

### Amending editome data

With an enlarged data set of editome references several cases became evident where editing sites may have been missed in previous analyses or where unexpected “orphan” editing events reported previously are restricted to individual taxa. The enhanced output now displaying non-edited or non-editable codons combined with the red highlighting of known editing events in the references facilitates interpretation of the results in the commons tab. For example, the presence of editing site ndhHeU505HY in phylogenetically distant taxa including *Amborella, Anthoceros, Cocos* and *Hevea* (red) and conservation of a genomically encoded tyrosine in other taxa (black; Fig. 2c) strongly suggested that the editing event was missed in the early *Cucumis* transcriptome study [43]. We recently checked upon such cases extensively in *Cucumis* confirming this and several other candidate sites to extend its chloroplast editome [40]. Here, we took the opportunity to selectively also investigate other cases, such as rps2eU134TI in *Atropa belladonna* (Fig. 2d) by independent cDNA analyses and indeed found that many such sites have apparently been overlooked in the previous transcriptome studies. Altogether we already confirmed 56 additional events of RNA editing in 10 species by our independent cDNA analyses (Additional file 2). The newly confirmed events of RNA editing now identified were incorporated into the updated PREPACT 3.0 references.

### Less conserved RNA editing sites and shifts in amino acid conservation

In some cases, it becomes apparent that a shift in amino acid conservation has obviously affected RNA editing sites during plant evolution. The *rps2* gene is a case in point, exemplarily shown for the *Atropa belladonna rps2* query (Fig. 2d). Editing of a serine codon in position 248 is fundamental in several dicot angiosperms to convert it into a leucine codon conserved in all taxa. In contrast, editing rps2eU107SF in the hornwort *Anthoceros* appears to reflect an ancestral state to reconstitute a conserved phenylalanine (F) codon in algae, liverworts, mosses and ferns (here represented by *Chara, Apopellia, Physcomitrella* and *Adiantum*) but not in the angiosperms, which lack editing and retain the genomically encoded serine codon. Editing event rps2eU134TI converting a threonine into an isoleucine codon is among those now to be added to the *Atropa* chloroplast editome (and the ones of *Oryza* and *Zea*) upon our re-investigation of cDNAs (Additional file 2). At the same time, this site is a further example for an editing event where another, but chemically similar, amino acid – in this case leucine (L) – is present at the corresponding position in early-branching taxa (Fig. 2d).

A similar case is the hornwort RNA editing atpIeU158SL serving to reconstitute a leucine codon conserved as a plesiomorphy in the early-branching plant lineages whereas a serine codon remains unaltered in the flowering plants (Fig. 2e). These are interesting cases of differential conservation of RNA editing sites and amino acid signatures, possibly indicating functional protein adaptations during evolution.

In few cases, codon changes introduced by RNA editing in the chloroplast seem erratic. The case of the “orphan” editing psbZeU11AV introducing a valine codon exclusively in the *Selaginella psbZ* transcript (Fig. 2f) is an example where a genomically encoded alanine is present and retained in all other references. Cases like these, in which chemically similar amino acids are exchanged, may reflect tolerable mis-firings of the editing machinery in taxa like the lycopyhtes where editing is particularly abundant. More complex are the divergent editing patterns even among angiosperms alone, like in the case of *ndhG* (Fig. 2g). Whether such cases reflect less conservation of the individual protein subunits, and accordingly of editing, or may rather indicate adaptations to interaction partners in the protein complexes, here possibly associated with a loss of *ndhG* editing in the Solanaceae (*Atropa* and *Nicotiana*), remains to be seen. While edit ndhGeU332SL in *Anthoceros* (Fig. 2g) could be an orphan edit like psbZeU11AV in *Selaginella* (Fig. 2f), this edit is in fact shared with *Selaginella* and may rather reflect conservation of a leucine in the early-branching land plants (not shown). Examples like these emphasize the importance of taxonomically diverse editome data sets.

### New features for multiple sequence comparisons

**Fig. 3:**
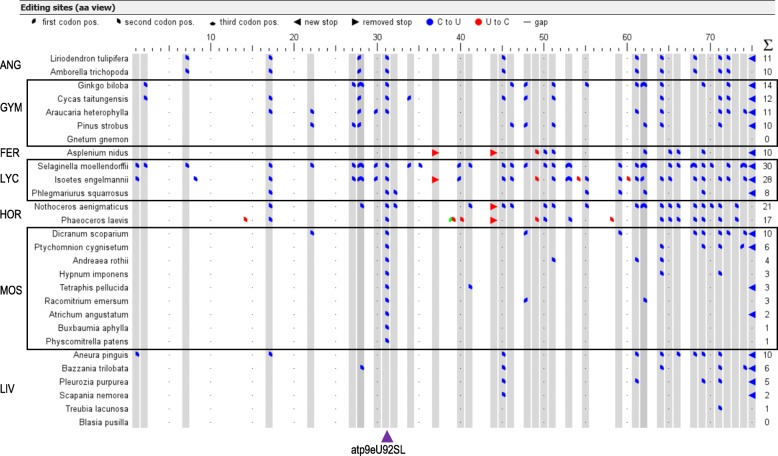
The new “pie-chart” mode for graphic display of RNA editing patterns allows to distinguish RNA editing events in first, second and third codon positions. Three sections (upper left, upper right and lower third) of a circle symbolize codon positions 1, 2 and 3, respectively. Forward and backward arrowheads indicate stop codons removed or introduced by RNA editing (see legend on top). In the query form (Additional file 1) users may adjust symbol sizes and different colours for C-to-U (here: blue) or U-to-C (here: red) RNA editing events and a threshold for highlighting edits shared between taxa by grey background shading (here: 2). Individual graphic displays are produced for each reference in the query, here exemplarily shown for the *Marchantia polymorpha* reference in the mitochondrial *atp9* example (Additional file 1). Editing site labels are indicated upon mouse-over at the editing symbols. Boxes and three-letter-acronyms are here added to designate the seven major plant clades: angiosperms (ANG), gymnosperms (GYM), ferns (FER), lycophytes (LYC), hornworts (HOR), mosses (MOS) and liverworts (LIV). The purple arrow at the bottom points to editing event atp9eU92SL conserved among many taxa and for which PpPPR_98 has been characterized as an editing factor in *Physcomitrella* (see Fig. 5a)

Aside from its BLASTX mode to identify coding regions and candidate RNA editing sites de novo in uncharacterized organelle sequence queries, PREPACT offers analyses of multiple sequence alignments for comparative analysis of RNA editing. The “cDNA” and the “alignment prediction” mode are intended for comparative analyses and graphic display for a set of homologous sequences including one or multiple references. We here demonstrate the new functionalities using aligned sequences of the small mitochondrial *atp9* gene for a phylogenetically wide sampling as an example for the alignment prediction mode (Additional file 1). Uploading a multiple-sequence FASTA file now displays the names of all individual sequences, which can be re-sorted in order, dragged-and-dropped between the collection of references and entries for prediction or can be individually deleted (Additional file 1). When multiple references are used, the output is organized into separate tabs for the individual references plus the comparative commons tab (if selected), analogous to the BLASTX mode (Additional file 3). We have now introduced the “pie chart” mode for more informative graphic display with three sections of a circle distinguishing editings in the three different codon positions (Fig. 3). Silent editing events in 3rd codon positions (and 1st position leucine YUR codons) is of relevance for the cDNA analysis mode only (not shown). The *atp9* example reflects several cases where simultaneous editing in codon positions 1 and 2 is needed to reconstitute conserved codons (codons 28, 53, 62 and 68) in the heavily editing lycophytes and gymnosperms. RNA editing frequencies vary significantly in each plant clade. The *Physcomitrella patens* RNA editing event atp9eU92SL affecting *atp9* codon 31, which we will also discuss below in the context of the new TargetScan feature, is shared by all other mosses, the hornworts, lycopyhtes, three gymnosperms and the two angiosperms in our example (Fig. 3).

### The EdiFacts module

**Fig. 4:**
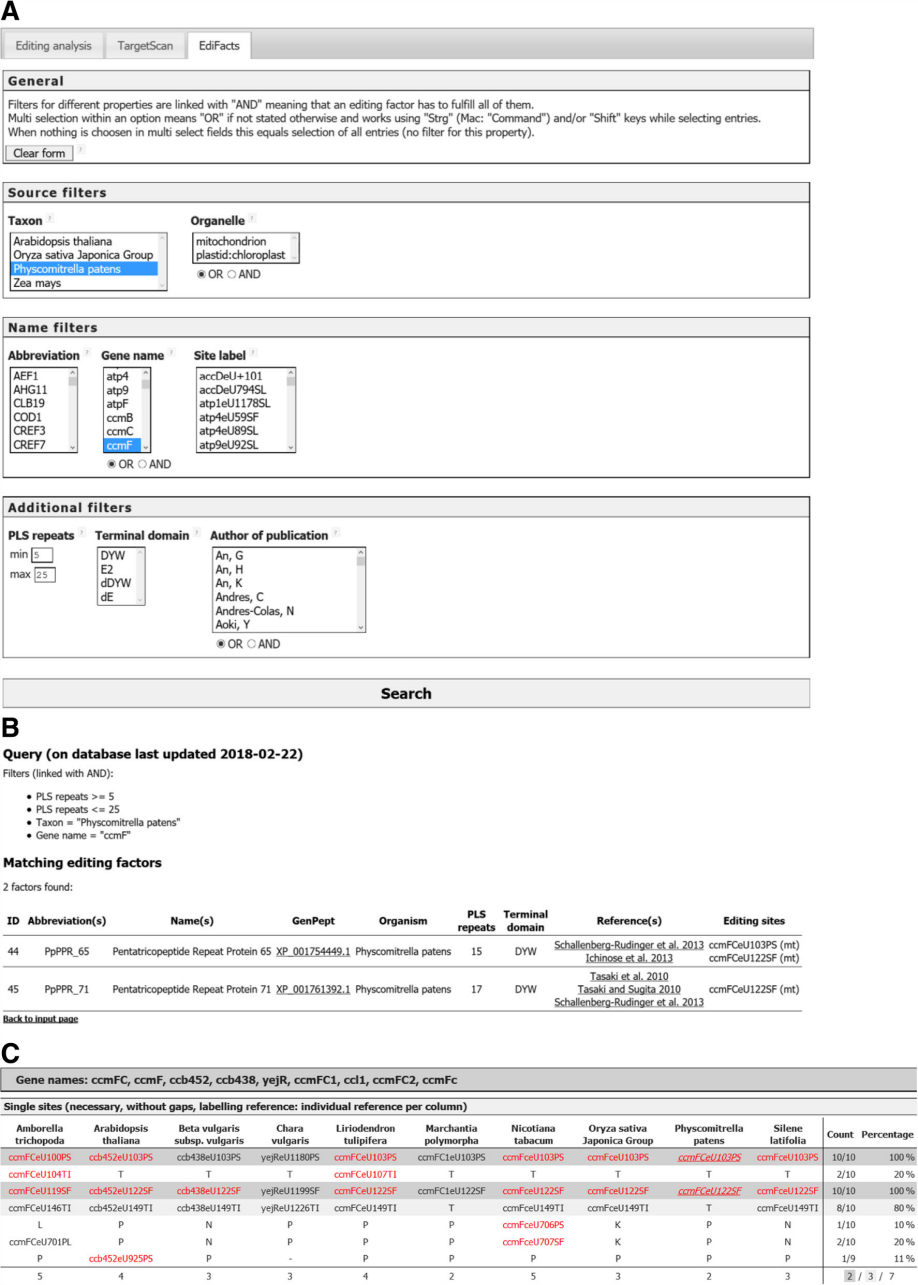
**a** The query form of the EdiFacts database module. Users may select to search for species, organelle, editing factor name, gene, editing site, factor-specific features such as length of the PPR arrays or carboxyterminal domains or authors of the corresponding publications to query the database. Boolean (AND/OR) logic can be adjusted where appropriate. The example shown reflects the search for characterized editing factors affecting the *Physcomitrella patens ccmFC* gene. **b** The EdiFacts output for the query shown under A retrieves *Physcomitrella* editing factors PPR_65 and PPR_71. Direct links to the respective protein sequences and literature reports are provided. **c** The PREPACT commons output highlights editing events ccmFCeU103PS and ccmFCeU122SF in *Physcomitrella patens* by italics and underlining to indicate that editing factors have been characterized for these RNA editing events. Clicking on these sites links to the entries for PPR_65 and PPR_71 in the EdiFacts database as shown under B. The *ccmF* homologues are notoriously complex owing to independent disruptions into separate ORFs and alternative gene names, which is accounted for by synonymizing in the PREPACT references (see output header). Shown is the example for the *Physcomitrella patens ccmFC* gene as a query run against the 10 selected references given in the header in BLASTX mode (see Fig. 1). The new “or reference site” option allows to include documented rare, unexpected or “orphan” editing events – in this example in *Amborella, Liriodendron, Nicotiana* and *Arabidopsis* – although below the overall default threshold level of 70% for display

As of writing of this manuscript, more than 70 nuclear-encoded RNA editing factors targeting specific chloroplast or mitochondrial RNA editing sites in plants have already been characterized. These site-specific RNA-editing factors are “PLS-type” PPR proteins with carboxyterminal E1, E2 and DYW domains. We have summarized the available information on the hitherto known editing factors as individual entries in “EdiFacts”, a database and query extension to PREPACT (Fig. 4). EdiFacts includes information about species, target genes and editing sites in the two organelles and links to the respective editing factor protein sequences and the corresponding literature reports. All data can be queried with Boolean AND/OR logic with options to choose multiple entries in fields where appropriate. Optional query restrictions can be made for the number of repeats in the PPR arrays, carboxyterminal protein domains or authors of the corresponding publications (Fig. 4a). This is exemplarily demonstrated with a query for “*Physcomitrella patens*” AND “*ccmF*” (Fig. 4a). This search retrieves the EdiFacts entries with ID 44 and 45 (Fig. 4b), corresponding to the characterization of *Physcomitrella patens* PPR proteins PPR_71 and PPR_65 [66, 67].

If a characterized factor is known for a given editing site, the PREPACT output of that editing event is now highlighted with italic font and underlining, and dynamically cross-linked to the respective EdiFacts entry as exemplarily demonstrated for the two *Physcomitrella ccmFC* editing sites (Fig. 4c). This allows users not only to identify candidate sites of editing where orthologous editing events have been seen in other taxa (red font), but immediately also reveals the information on co-factors when already known (italics and underlining). The two mitochondrial RNA editing sites, ccmFCeU103PS and ccmFCeU122SF, are widely conserved in the plant kingdom, but pre-edited as serine and phenylalanine codons in the (non-editing) alga *Chara* and the liverwort *Marchantia* in our sampling. Our *ccmFC* example (Fig. 4c) also illustrates further examples of phylogenetically restricted (ccmFCeU104/107TI in *Amborella* and *Liriodendron*) or “orphan” RNA editings ccb452eU925PS in *Arabidopsis* and ccmFCeU706PS and ccmFCeU707SF in *Nicotiana*.

### The TargetScan module

**Fig. 5:**
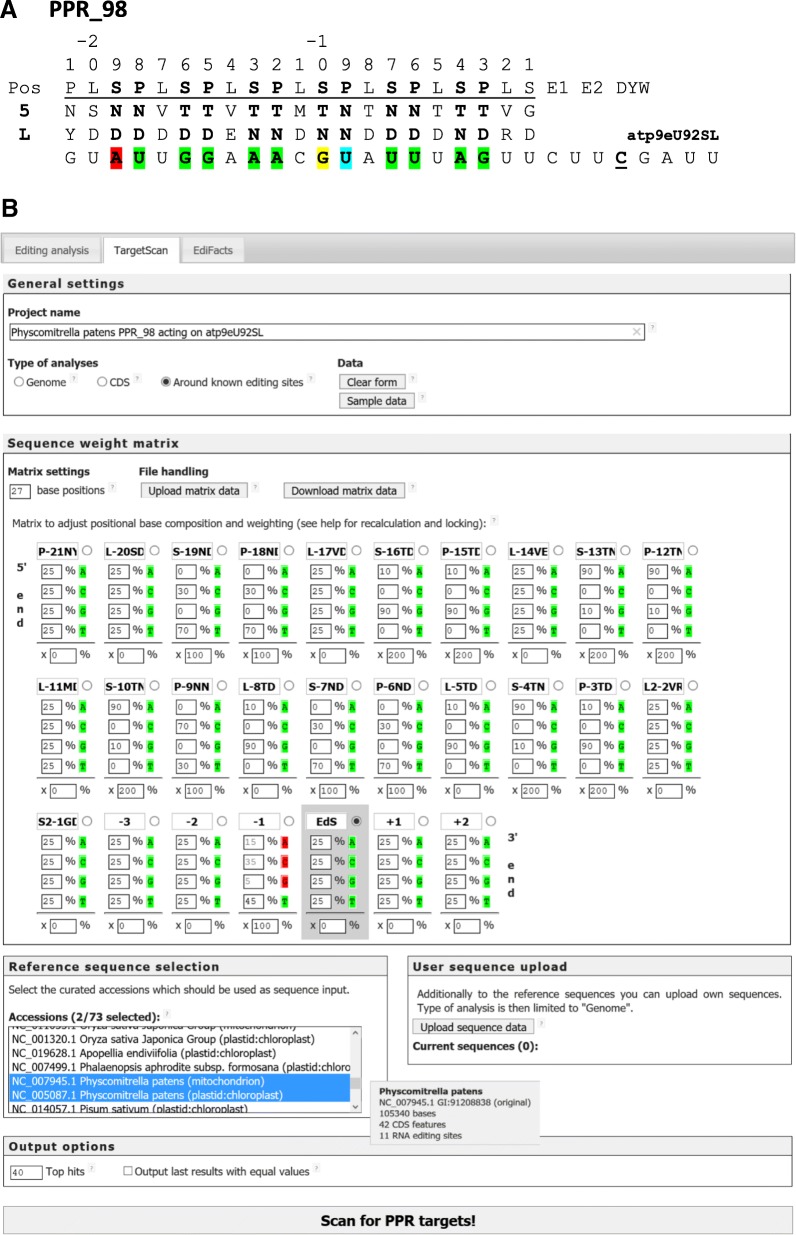
**a** The example of PPR_98 targeting the atp9eU92SL editing site in *Physcomitrella patens*. PPR_98 is a typical DYW-type PPR protein with 21 canonical PLS repeats. Nine of its P- and S-type PPRs (bold) perfectly follow the core RNA recognition rules (green shading) with amino acids T or N in position 5 to select for purines vs. pyrimidines, respectively, and amino acids D or N in position L (“Last”) to select for keto vs. amino bases in the target RNA. Binding properties of L-type PPRs and the divergent terminal “S2-type” PPR linking to the E1 domain are not understood. We here suggest an annotation with reverse numbering of PPRs starting with the last repeat and indicating the amino acids in positions 5 and L as shown in the PPR labels under B. In three cases, nucleotides other than expected are juxtaposed with the target: A instead of the expected U in S-19 (transversion, red shading), G instead of A in S-10 (purine transition, yellow shading) and U instead of C in P-9 (pyrimidine transition, blue shading). **b** The TargetScan interface allows to set a length for a weighted nucleotide query (top left) which then becomes available for definition and adjustment in all positions (middle). An additional overall weighting can be given below for each position. The example shown reflects the deduced atpeU92SL target shown in A. Upon assigning a value (0–100%) for a given nucleotide identity it can be temporarily “locked” by a click (switching green to red), allowing for further adjustments of the remaining identities, which automatically add up to 100% for a given position. This is exemplarily shown for position − 1 where the empirically observed pyrimidine bias is here reflected by arbitrary weights fixed for A (15%), C (35%) and G (5%) to automatically set 45% for T. The example shows arbitrary adjustments for P- and S-type PPRs assuming a 90 vs. 10% selectivity between purines (T/S + N for A vs. T/S + D for G) and a 70 vs. 30% selectivity for pyrimidines (N + N/S for C vs. N + D for U). Positions identifying purines receive a double weight (200%), pyrimidines and position − 1 receive 100% weight. Selecting “Around editing sites” (top) allows to fix any site to be a documented edit within the PREPACT references (‘EdS’) by selecting a radio button. The *Physcomitrella patens* mitochondrial and chloroplast editome references are selected for querying. Mouse-over gives information on the respective reference, here shown for the *Physcomitrella* mtDNA. As an alternative to PREPACT’s editome references, users may upload alternative sequences for querying

A new module TargetScan has been added to allow position-weighted querying of the PREPACT organelle reference data sets or of user-uploaded sequences for sequence motif matches (Fig. 5). The TargetScan interface allows users to define an oligonucleotide sequence motif with individual weighting of base preferences for A, C, G and T(U) using integers that automatically add up to 100 (%). Accordingly, a weighting of 25–25–25-25 reflects no nucleotide selectivity (equals “N” ambiguity) whereas 0–40–0-60 would, for example, reflect a strong selectivity for pyrimidines with a slight preference of T over C. Single input weights can be locked by clicking onto the respective nucleotide (switches from green to red background), distributing the remaining percentage evenly across the non-locked variants (Fig. 5b). Matrix input may be saved by download and re-uploaded. Users may select any combination of PREPACT references and/or other uploaded data for querying, hence allowing to scan for sequence targets across arbitrarily selected organelle references. Additional options allow to restrict the search for sequence targets within coding sequences or to regions around known editing sites (only in annotated references). While TargetScan may be helpful for diverse other issues, the latter options are mainly intended to identify and rank candidate targets of PPR-type editing factors.

We here exemplarily demonstrate the use of TargetScan for the *Physcomitrella patens* editing factor PPR_98 (Fig. 5a), which has been characterized as the specificity factor binding to the target sequence upstream of editing site atp9eU92SL (see Fig. 3) in vivo and in vitro [67, 68]. PPR_98 is a canonical (PLS)_7_-type PPR protein with a terminal DYW domain.

At the core of the PPR-RNA binding code [28] are position 5 selecting purines vs. pyrimidines with amino acid residues T (or S) vs. N and position L (‘Last’) selecting the keto nucleotides G or U vs. the amino nucleotides A or C with amino acids D vs. N in the PPRs (Fig. 5a). The first selection mechanism for purines vs. pyrimidines appears to be stronger and so is the distinction between the two purines as compared to the two pyrimidines. Moreover, the suggested code only fits the P- and S-type but not to the L-type PPRs, the functions of which remain to be explored. Accordingly, we arbitrarily weighted 90 vs. 10 for purine and 70 vs. 30 for pyrimidine selection in the canonical T/S + D, T/S + N and N + N/S or N + D-carrying P- and S-type motifs with pyrimidine recognition weighted as 100% and purine recognitions weighted as 200%. Additional weight was given to the position immediately upstream (− 1) of the cytidine editing target, here set arbitrarily to 15, 35, 5 and 45% for A, C, G and T (Fig. 5b) based on empirical observations that purines, and especially guanosines, occur only rarely upstream of an editing site (see also the new investigations outlined in the following chapter). All other positions were weighted 0% (Fig. 5b). In the case of PPR_98 and its target, nine PPRs fit perfectly to the above concept, whereas the binding code would suggest other nucleotide preferences for one P- and two S-type PPRs (Fig. 5a).

**Fig. 6:**
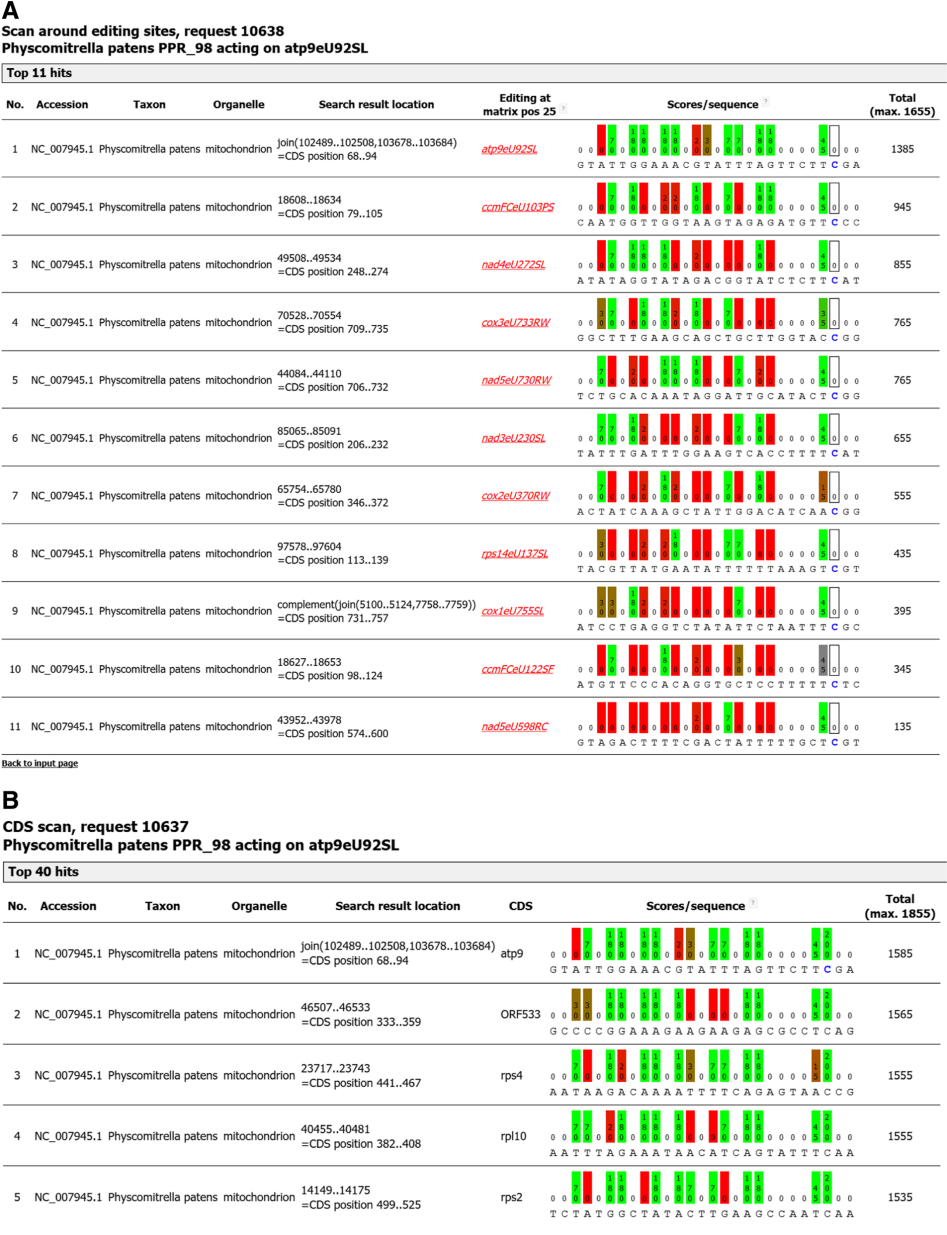
**a** The hit ranking is shown for the output of TargetScan with the query settings shown in Fig. 5b to search around the known *Physcomitrella* chloroplast and mitochondrial editing sites. Edited cytidines in the list of candidate sequence targets are shown in blue. Individual nucleotide positions are colour shaded from green (“matching”) to red (“mismatching”) for easy interpretation. Total scores are the sum of individual position scores multiplied by their weight and the maximum score is indicated top right. The true mitochondrial PPR_98 target upstream of atp9eU92SL receives a top hit scoring of 1385 and is set apart from the other editing sites receiving lower scores in the range between 135 and 945. Editing factors are known for all *Physcomitrella* editing sites as indicated by italics and underlining, linking to the respective EdiFacts entries. **b** Top ranking hits with the same settings as shown in Fig. 5 but now searching in all mitochondrial coding sequences (CDS) and adding a score of 200 for alternative candidate cytidine targets to place them on top of the ranked output (max. score now 1855 instead of 1655)

With the simple arbitrary weights outlined above, the correct RNA editing target of PPR_98 is identified with a top score of 1385 (calculated from the sum of all positions with their individual scores multiplied by their weight) set apart from the second-best hit (the ccmFCeU103PS editing site scoring 945) when scanning upstream of all *Physcomitrella* chloroplast and mitochondrial RNA editing sites (Fig. 6a). Such a ranking of matches among known editing sites is certainly helpful to identify best candidate targets of uncharacterized PPR proteins when editing sites are known. However, any amendments to the PPR-RNA recognition mechanisms must always explain why any similar transcribed sequences (at least in the same organelle) are not targeted. Extending the above search to all *Physcomitrella* mitochondrial coding sequences (CDS) with the above settings (plus an added arbitrary 200% for C at the candidate editing position to place potential cytidine targets top of the list) still identifies the atp9eU92SL editing site as the top-ranking target (Fig. 6b). However, the next-best candidate cytidines for editing in *orf533, rps4, rpl10* and *rps4* receive nearly as good scorings for upstream binding using the simple scoring scheme outlined above. Such cases warrant for (i) re-inspection for RNA editing at such candidate alternative target sites, (ii) testing for different binding weights of the different PPR motifs and target positions and (iii) subsequent testing with recombinant proteins in model systems like *Physcomitrella* and *Arabidopsis*. Evidently, any TargetScan query motif has a different a priori probability to identify top-scoring hits in different organelle genomes depending on nucleotide composition (mainly GC content). However, any results with identical scores identified in different genomes should indicate equally good matches, e.g. as likely targets for a given PPR protein like in the example discussed above.

### Using TargetScan to explore the immediate editing site environments

**Fig. 7:**
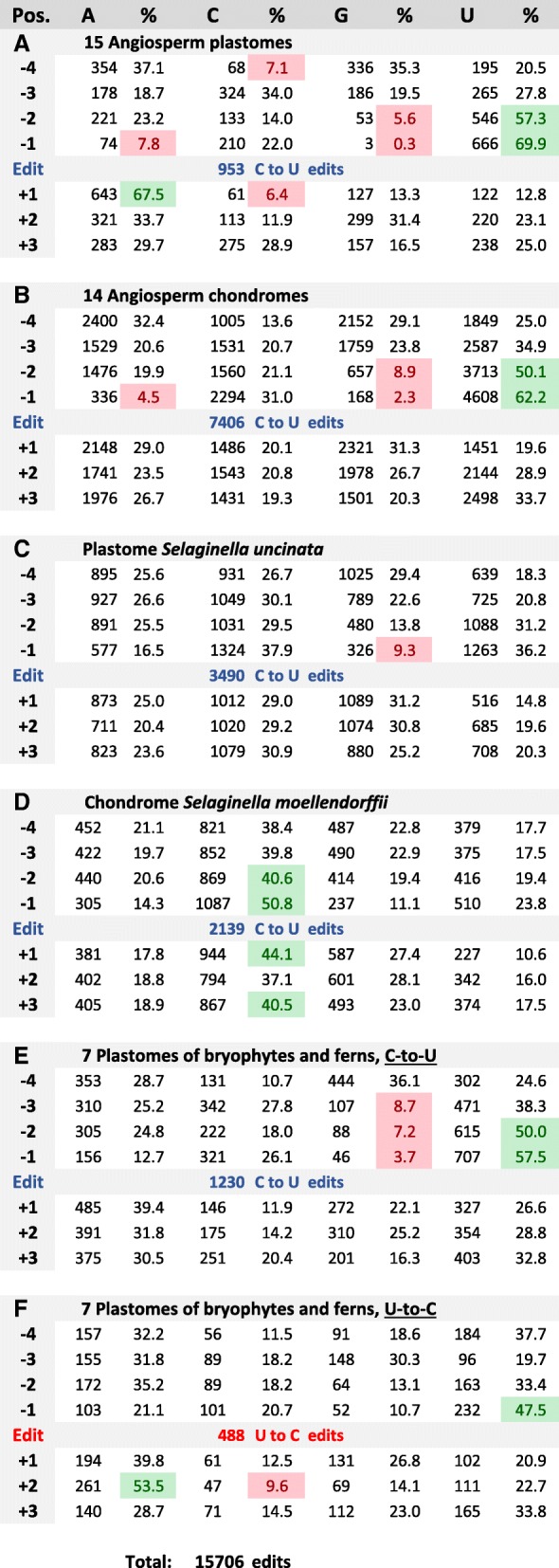
Results for querying positions − 4 to + 3 around editing sites in six different editome datasets **a**-**f** as indicated. The numbers of respective nucleotide identities in a given position are given, followed by the respective percentages. Colour shading indicates nucleotide frequencies lower than 10% in red and higher than 50% in green. The numbers of C-to-U edits (blue) and U-to-C edits (red) are given for each data set, totalling altogether 15,706 in these selected PREPACT reference editomes

The growing amount of complete and reliable organelle editome data now also allows to identify potential nucleotide bias in the immediate sequence environment of editing sites. These RNA positions are currently not assumed to be targets for recognition by PPRs, but nucleotide preferences could result from close interactions with the downstream E1, E2 and DYW domains of editing factors. We here investigated the immediate sequence vicinity of editing sites in positions − 4 to + 3 for nucleotide bias in six different organelle editome data sets now included in PREPACT: angiosperm plastomes, angiosperm chondromes, the *Selaginella uncinata* plastome, the *Selaginella moellendorffii* chondrome and the available chloroplast editomes of bryophytes and ferns, here considering C-to-U and U-to-C editing events separately (Fig. 7).

As an alternative to more demanding script programming approaches, users may employ simple weight matrices in TargetScan for such and similar purposes. Using arbitrary weightings for the four different nucleotides in each position (e.g. A:40, C:30, G:20, T:10), one can quickly obtain sorted results for each nucleotide identity as exemplarily shown for position − 1 in the angiosperm plastome example (Additional file 4). Compiling the data for positions − 4 to + 3 reveals striking differences for the six different editome sets concerning nucleotide bias in the positions surrounding the editing sites (Fig. 7).

It had been noted early that guanosines occur only very rarely upstream of edits. We now find that the bias against G is most pronounced in angiosperm chloroplast editomes (Fig. 7a) with only 0.3% G (and only 7.8% A) vs. a strong preference for U (69.9%) in position − 1. A similar picture strongly avoiding purines directly upstream of edits also emerges for the angiosperm chondromes (Fig. 7b) with only 2.3% G and 4.6% A. Likewise, a similar bias is also found in the bryophyte and fern chloroplast editomes, but significantly less so for the U-to-C editing events co-existing with C-to-U editing in these taxa (Fig. 7e and f). Surprisingly, for the reverse U-to-C editing sites in the hornwort and fern plastomes (Fig. 7f), another strong nucleotide bias in position + 2 behind the editing site (53.5% A vs. 9.6% C) even outnumbers the less pronounced bias in position − 1. While still biased against G, cytidines even dominate over uridines in position − 1 in the GC-rich organelle genomes of *Selaginella* (Fig. 7c-d).

Notably, our survey identifies yet further strong bias in other positions. Most prominent is the strong bias against C (7.1%) in position − 4 and the dominance of A (67.5%) vs. C (6.4%) in position + 1 for the angiosperm chloroplast editomes (Fig. 7a). These preferences are less pronounced, but recognizable, in the bryophyte and fern plastomes (Fig. 7e and f), but not in the angiosperm chondromes (Fig. 7b), possibly indicating slight differences between the editing machineries in the two endosymbiotic organelles.

Moreover, the U vs. G bias in position − 1 is now also becoming evident for position − 2 both in the chloroplast and in the mitochondrial editomes of angiosperms (Fig. 7a and b) and likewise also for C-to-U editing in bryophyte plastomes (Fig. 7e). In position − 3, however, only bryophytes and ferns, but not angiosperms, show a clear uridine over guanosine bias. Again, the latter findings cannot be generalized for the exceptionally GC-rich organelle genomes of *Selaginella* (Fig. 7c and d).

## Discussion

The functional extensions of PREPACT presented here serve different purposes for the analyses of “plant-type” C-to-U and U-to-C RNA editing in organelles. The upgrade of PREPACT’s repository of organelle editomes extends its functionality in the prognosis and comparative analysis of RNA editing. The success of RNA editing prognoses can be expected to increase with a wider sampling of references, especially of taxa more closely related to the query sequence taxon. Denser taxon sampling will help to identify both overlooked and orphan cases of RNA editing in individual taxa, as we have here shown exemplarily. Given the likely ever-growing set of editome information, a future update of PREPACT aims to include an option to select sets of references based on higher taxonomic ranks. In the future, we also hope to include additional complementary information from the literature for individual editing sites such as e.g. conflicting reports, strain variabilities or variable editing frequencies depending on development. Especially regarding a better understanding of the PPR-RNA recognition code discussed below, it is particularly important that editome analyses are correct and complete, i.e. without false positives and without actual editing events being missed. We found that some previous studies of organelle editomes based on early RNA-seq data require re-analyses. Meantime, suggestions for adequate design and improved analysis of such studies have been made [69, 70].

The implemented editome references will make PREPACT an expanding database of reported C-to-U and U-to-C RNA editing sites. Moreover, with the increasing number of discoveries of C-to-U RNA editing also outside of plants [24, 26], PREPACT will likely extend its scope in the future to also include yet more non-plant taxa. We hope to be able to provide timely updates of PREPACT and further reference editomes in the future.

Equally important, we have now integrated data on the increasing number of PPR-type editing factors that have been functionally characterized in the new database module EdiFacts. Accordingly, the user directly obtains information that an editing factor has been characterized in a reference taxon for a predicted editing position. This aspect is of considerable interest given the co-evolution of organelle editing sites and their nuclear-encoded specificity factors. A PPR-RNA binding code has been proposed, which is currently being refined and experimentally tested [28, 30, 31, 71, 72]. The proposed PPR-RNA recognition code offers an exciting field for bioinformatic and subsequent reverse genetic testing of proposed interactions between PPR arrays and RNA targets. However, other protein features outside of the immediate PPR-RNA interaction surface such as the immediate nucleotide environment of the editing sites [73] need to be taken into account, too. Future amendments, refinements and additional assumptions on how the RNA recognition factors recognize their target(s) need to be tested against the actual in vivo situation, i.e. all alternative transcriptome targets in a given organelle. We hope that the here described TargetScan module will be of help towards this issue. With respect to accompanying molecular studies, the moss *Physcomitrella patens* now occupies a unique position after completing the assignment of all its organelle RNA editing sites to specific cofactors [67, 68, 74]. Given its features as a genetic model organism it may be particularly attractive for transformation with mutated versions of RNA editing factors. For example, it has been shown that the terminal (and likely cytidine deaminase) DYW domains of different editing factors are exchangeable in some [75], but not in other cases [76], indicating at least a partial preference for their native targets, likely depending on the immediate sequence environment of the cytidine to be edited. Much further understanding is needed to adequately tune the (here arbitrarily selected) weights for those positions currently understood to contribute to sequence recognition (the P- and S-type PPRs) and ascribing proper weights to those elements (like the L-type PPRs, the E1, E2 and the DYW domains), for which a contribution to confer sequence specificity has yet to be elucidated.

The manual identification of potentially relevant amino acid positions from the loosely conserved P-, L- and S-type repeats of PPR proteins is tedious and cumbersome. The TPRpred tool served as a publicly available bioinformatic service for de novo identification of tetratricopeptide (TPR) and pentatricopeptide (PPR) repeats [77]. A new WWW-service (initially under www.plantppr.com, now available at ppr.plantenergy.uwa.edu.au) allows to distinguish the P-, L- and S-type PPRs of plant PLS-type editing factors specifically after carefully reconsidering the domains of plant-type PPR proteins [78]. An automatic extraction of the key residues like PPR positions 5 and L and their direct translation, e.g. via appropriate lookup-tables, for direct input into the new TargetScan of PREPACT is a future goal. Independent of this approach, the mutual assignment of editing sites and editing factors as implemented with EdiFacts will hopefully already now help to further explore the yet enigmatic co-evolution of organelle RNA editing sites and their nuclear co-factors.

## Conclusions

Over the recent years, research on plant-type RNA editing has extended to the characterization of the specific, RNA-binding pentatricopeptide repeat (PPR) protein factors addressing individual editing sites in the endosymbiotic organelles. We have accordingly extended our WWW service PREPACT to include information on PPR-type editing factors in an additional database module EdiFacts. As a further extension of PREPACT, the new TargetScan module allows to search for position-weighted motifs in the now extended reference editome set of PREPACT or in user-defined references. The novel feature now implemented in version 3 of PREPACT should be of use to integrate information of RNA editing sites and corresponding specificity factors and help to further elucidate how PPR-type editing factors recognize their respective RNA targets.

## Methods

The core functional implementation of PREPACT using PHP and MySQL has been described earlier [38, 39]. Basic functions have been revised to yield higher performance and to cope with growing query complexity. This included consistent translation of different sequence/feature numbering schemes on a global and local scale to be able to match information in partial hits and globally numbered features. The internal GenBank engine has been extended to also handle remote locations (in other accessions) and partial CDS features with annotated editing sites locally as well as in the remote part. This was necessary to also deal with complex genomes split across multiple accessions in parallel with trans-splicing as e.g. in the *Amborella trichopoda* mitochondrial DNA. The reference tabs of the BLASTX output (see Fig. 2) now offer an option for download of the individual references in a GenBank-style flat file format including the standardized annotation of RNA editing sites with the additional “RNA_editing” feature we had introduced previously [39].

The user interface has been improved mainly on the sequence upload/handling side via integration of additional JavaScript features with the help of jQuery (https://jquery.com/) and jQueryUI (https://jqueryui.com/) libraries as well as additional jQuery extensions “File Upload” (https://blueimp.github.io/jQuery-File-Upload/) and “Add Clear” (https://github.com/skorecky/Add-Clear).

EdiFacts is an addition to the relational database with data collected manually from publications. New items are continually identified by routine literature searches, journal publication alerts and journal scanning services such as “PubCrawler” [79] using appropriate key words. Literature references are downloaded, parsed and stored locally for search purposes and linked to respective external NCBI PubMed and protein source entries. Editing sites affected by listed factors are referenced in the “RNA_editing” feature introduced in PREPACT2 [39] using a “db_xref” qualifier. This internal crosslink is used for highlighting editing sites with known editing factors in the “commons” output. The EdiFacts input form is the graphical representation of the internal query builder which translates various combinations of selected filters/options into efficient MySQL queries combining all available data.

The TargetScan module is comparing the user-defined weight matrix in a sliding window approach to the selected sequences or sequence parts extracted from the internal GenBank database. As such, TargetScan is a custom-made and easy-to use alternative to more sophisticated motif identification algorithms such as FIMO [80] or PWMscan [81]. Scores for each sub-sequence are calculated by multiplying the base value (percent) with the position weight and summing up. Results are ranked by descending score down to a certain number of results or optionally to all results with the same score after this number of results to avoid arbitrary cut-offs of equally good matching sub-sequences. In the output individual base stretches are listed with their position/features according to the selected mode and single base scores are colour coded from green (maximum score at this position = perfectly matching) to red (minimum score at this position), with mixed colours in between. Positions with no weight are excluded from colour coding to have less clutter. Editing sites are highlighted in the sequence in blue (C-to-U) or red (U-to-C) respectively. To be in line with other sequence features, the selection of sub-sequences for searching in different modes (“Genome”, “CDS”, “Around editing sites”) is internally implemented as an extension to the GenBank format defining “Search_range” and “Search_result” as GenBank features.

For detection of previously overlooked RNA editing sites, individual chloroplast references were run against all other available reference editomes. Strongly predicted editing sites (i.e. with a ‘commons’ score of at least 80% or at least one edited reference species) previously not reported not to be edited were rechecked in selected cases (Additional file 2). To that end, plant material was obtained from the Bonn University Botanic Garden Bonn and RNA was prepared by the CTAB method, the TRI Reagent Protocol (Sigma Aldrich) or with the NucleoSpin® Plant RNA II Kit (Macherey-Nagel). Subsequently, cDNA synthesis was performed with Revert Aid First Strand cDNA Synthesis Kit (Thermo Fisher) using random hexamer primers. The relevant regions were amplified by RT-PCR with gene-specific primers and products recovered from agarose gel with NucleoSpin® Extract II Kit (Macherey-Nagel). PCR products were sequenced directly after gel elution or after cloning into pGEM-T Easy (Promega).

## Additional files

Additional file 1: Multiple sequence input. An example for multiple query and reference sequence input in PREPACT’s alignment modes as discussed in the text. (DOCX 198 kb)

Additional file 2: Table of re-checked edits. Verification of additional RNA editing events previously overlooked in editome references. (DOCX 45 kb)

Additional file 3: Alignment prediction output. An example for the output of a multiple-query alignment for different references. (DOCX 79 kb)

Additional file 4: TargetScan of Editing environment. An example illustrating the use of TargetScan to identify nucleotide bias in the immediate environment of editing sites in position -4 to +3 as discussed in the text. (DOCX 87 kb)

## Abbreviations

CDS: Coding sequence
CTAB: Cetyl-trimethyl ammonium bromide
PPR: Pentatricopeptide repeat
PREPACT: Plant RNA editing prediction and analysis computer tool
