# Supplementary material for: Plant organelle RNA editing and its specificity factors: enhancements of analyses and new database features in PREPACT 3.0
Source: BMC Bioinformatics. 2018 Jul 3;19:255. doi: 10.1186/s12859-018-2244-9 (PMC6029061; doi:10.1186/s12859-018-2244-9)

**Additional file 1.**

The input for the “Alignment prediction” mode of PREPACT 3.0, here exemplarily demonstrated for an alignment of mitochondrial *atp9* sequences for a wide sampling of plants and algae. After upload of a FASTA alignment file, the individual sequences may be freely sorted and distributed within and between the reference (left) and prediction (right) panels by simple drag-and-drop. Sequence information can be retrieved by clicking on the arrows in front of the sequence name as exemplarily shown for the *Chaetosphaeridium globosum* sequence (top left). Info icons indicate messages regarding the import/upload available in the details. Individual sequences may be deleted by a click on the trash bin icon. The new “pie chart” option is selected to distinguish editing in the three different codon positions for display (see Fig. 3). The “variant grouping” option, which clusters query sequences with identical editing patterns, is turned off to retain the taxonomic order of sequences as manually defined in the input. Settings for the commons tab are analogous to the pre-settings for BLASTX mode (Fig. 1). Selected output of the analysis is shown in supplementary file 3 and in figure 3.


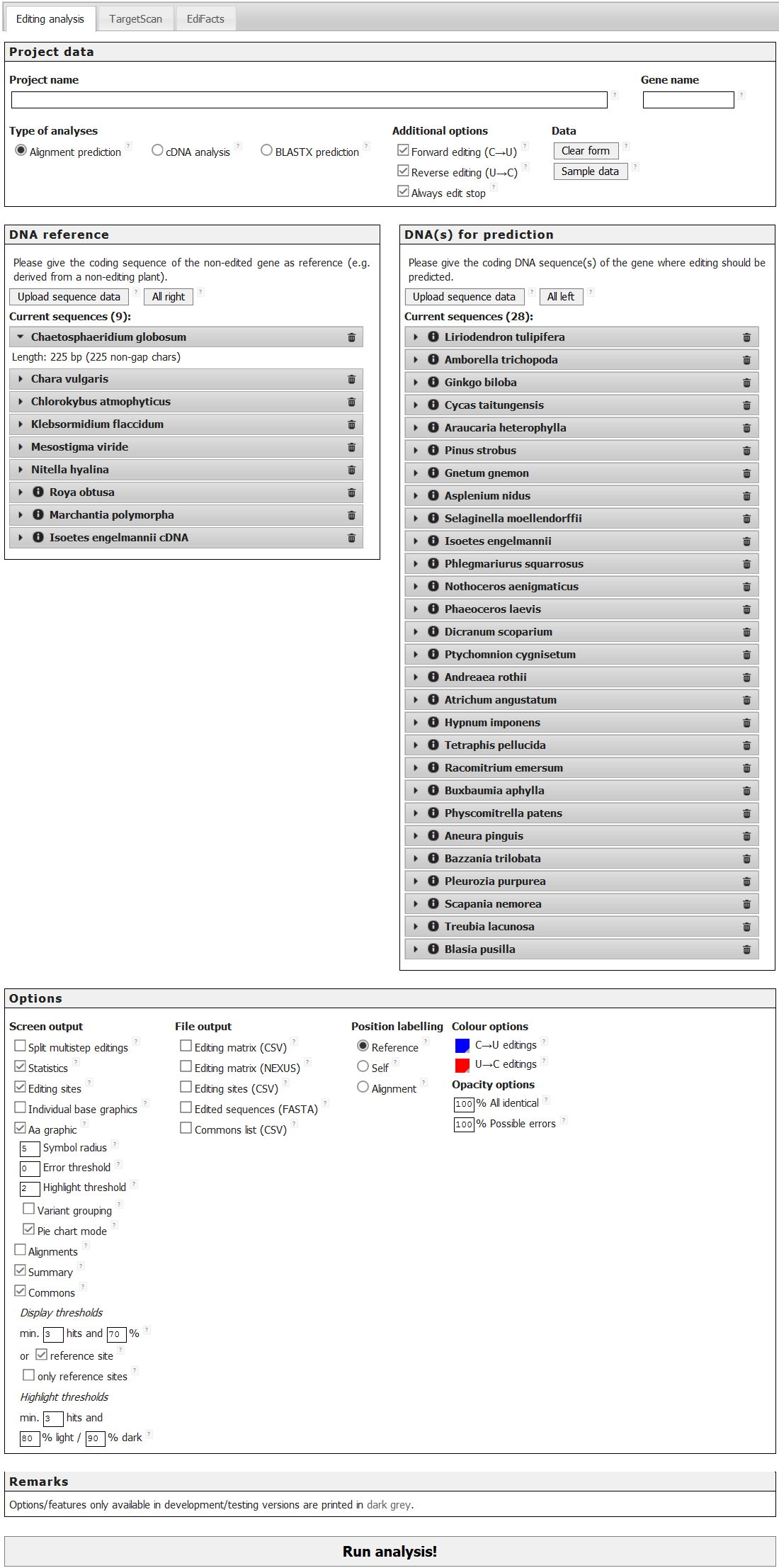

Supplement: Supplementary file 1 — Multiple sequence input. An example for multiple query and reference sequence input in PREPACT’s alignment modes as discussed in the text. (DOCX 198 kb) [file 12859_2018_2244_MOESM1_ESM.docx]
