# Supplementary material for: Plant organelle RNA editing and its specificity factors: enhancements of analyses and new database features in PREPACT 3.0
Source: BMC Bioinformatics. 2018 Jul 3;19:255. doi: 10.1186/s12859-018-2244-9 (PMC6029061; doi:10.1186/s12859-018-2244-9)
Supplement: Supplementary file 2 — Table of re-checked edits. Verification of additional RNA editing events previously overlooked in editome references. (DOCX 45 kb) [file 12859_2018_2244_MOESM2_ESM.docx]

**Additional file 2.**

Cross-checking angiosperm chloroplast references in PREPACT 3.0 identified several additional candidate sites of RNA editing possibly overlooked in previous transcriptome studies. Species abbreviations are derived from the first letter of the genus and the first three letters of the epithets of the PREPACT references (*Arabidopsis thaliana, Atropa belladonna, Cocos nucifera, Cucumis sativus, Gossypium hirsutum, Hevea brasiliensis, Nicotiana tabacum, Oryza sativa, Phalaenopsis aphrodite, Pisum sativum, Zea mays*). Editing nomenclature is as proposed earlier [65]. An arbitrarily chosen reference taxon is given for clarity of numbering in case of indels in the coding regions. The table summarizes cases where we checked on such sites with independent cDNA sequencing in at least one taxon. Capital letters indicate pre-edited or other codon identities, ‘ed’ indicates previously reported RNA editing, the hyphen (‘-‘) indicates that no homologue exists in the respective plastome, ‘no’ indicates that our study confirms absence of editing and question marks indicate open issues. The fields labelled ‘ed+’ report our independent confirmation of novel editing. Superscripts indicate that ndhEeU233PL^1^ is a change from a serine instead of a proline to a leucine codon in *G. hirsutum* and *H. brasiliensis* and that psbNeU29SF^2^ is accompanied by a silent downstream edit in position 30 in *C. nucifera*.

|  | Ref | **Atha** | **Abel** | **Cnuc** | **Csat** | **Ghir** | **Hbra** | **Ntab** | **Osat** | **Paph** | **Psat** | **Zmay** |
| --- | --- | --- | --- | --- | --- | --- | --- | --- | --- | --- | --- | --- |
| **accDeU1186HY** | Paph | C | Y | **no** | Y | Y | Y | Y | - | Y | Y | - |
| **accDeU1430PL** | Paph | L | L | ed | **ed+** | ed | L | L | - | ed | L | L |
| **accDeU1412PL** | Paph | A | Q | **ed+** | L | ? | L | L | - | ed | L | - |
| **atpAeU1342PS** | Abel | G | S | **no** | S | S | S | S | S | A | S | S |
| **atpAeU1390HY** | Abel | Y | Y | **no** | Y | Y | Y | Y | **no** | Y | Y | **no** |
| **atpIeU658LF** | Atha | F | ? | **no** | F | F | F | **no** | F | F | F | F |
| **ccsAeU970PS** | Atha | S | S | S | **no** | S | S | S | S | S | S | S |
| **clpPeU281TI** | Paph | I | V | I | I | I | V | I | **ed+** | I | V | I |
| **clpPeU508PS** | Atha | S | S | S | **no** | S | S | S | S | S | S | S |
| **clpPeU559HY** | Atha | ed | Y | ed | **ed+** | ed | ed | Y | Y | ed | Y | Y |
| **ndhAeU341SL** | Abel | **ed+** | ed | L | **ed+** | ed | ? | ed | L | - | ed | L |
| **ndhAeU566SL** | Abel | L | ed | ed | **ed+** | ed | ed | L | ed | - | L | ed |
| **ndhAeU961PS** | Hbra | S | **ed+** | ed | S | S | ed | **ed+** | S | - | S | S |
| **ndhBeU542TM** | Ghir | M | M | ed | **ed+** | ed | ed | M | M | - | M | M |
| **ndhBeU1112SL** | Acap | L | L | ed | **ed+** | L | L | L | L | - | L | L |
| **ndhCeU311PL** | Atha | L | L | **ed+** | L | L | L | L | L | - | L | L |
| **ndhCeU323SL** | Atha | L | L | **ed+** | L | ed | ed | L | L | - | L | L |
| **ndhDeU599SL** | Hbra | L | L | L | **ed+** | L | ed | L | L | L | L | L |
| **ndhDeU878SL** | Hbra | ed | ed | L | ed | ed | ed | L | ed | - | **ed+** | ed |
| **ndhDeU1298SL** | Ghir | L | **ed+** | L | L | L | L | **ed+** | L | - | L | L |
| **ndhDeU1310SL** | Ghir | L | **ed+** | ed | L | ed | L | **ed+** | L | - | L | L |
| **ndhEeU233PL** | Ghir | L | L | L | **ed+** | ed**^1^** | ed**^1^** | L | L | - | L | L |
| **ndhFeU290SL** | Ntab | ed | ed | ed | A | ed | **ed+** | ed | L | **-** | ed | L |
| **ndhHeU505HY** | Hbra | Y | Y | ed | **ed+** | Y | ed | Y | Y | - | ed | ed |
| **ndhJeU67HY** |  | Y | Y | C | Y | Y | Y | Y | ? | Y | Y | **no** |
| **ndhJeU128SL** | Atha | L | F | **ed+** | L | L | L | F | L | - | L | L |
| **ndhKeU65SL** | Atha | L | L | ed | **ed+** | L | ed | L | L | - | L | **ed+** |
| **ndhKeU497TI** | Atha | I | I | **no** | I | I | I | I | ? | - | I | **no** |
| **ndhKeU676Q*** | Atha | * | * | **no** | * | * | * | * | * | - | - | * |
| **petAeU91PS** | Atha | S | S | S | **no** | S | S | S | S | S | **no** | S |
| **petBeU418RW** | Atha | W | W | ed | **ed+** | ed | W | W | W | W | W | W |
| **petDeU481Q*** | Aang | * | * | **ed+** | * | * | **ed+** | * | * | **ed+** | * | * |
| **petGeU65SF** | Aang | F | F | F | **ed+** | F | F | F | F | F | F | F |
| **psaAeU1438LF** | Atha | F | F | F | **no** | F | F | F | F | F | F | F |
| **psaAeU1828RW** | Atha | W | W | W | **no** | W | W | W | W | W | W | W |
| **psaIeU79HY** | Psat | **no** | **no** | ed | **ed+** | Y | Y | **no** | Y | Y | ed | Y |
| **psbAEu854SF** | Atha | F | F | F | **no** | F | F | F | F | F | F | F |
| **psbBeU194SF** | Atha | F | F | F | **ed+** | F | F | F | F | F | F | F |
| **psbCeU529LF** | Atha | F | F | **no** | F | F | F | F | ? | ? | F | **no** |
| **psbFeU77SF** | Ghir | ed | F | F | **ed+** | ed | **ed+** | F | F | ed | ed | F |
| **psbJeU59PL** | Ghir | L | L | L | **ed+** | ed | L | L | A | L | V | L |
| **psbKeU5PL** | Atha | L | L | L | L | L | L | L | **no** | L | L | **no** |
| **psbKeU13LF** | Atha | F | F | F | **no** | F | F | F | **no** | F | F | **no** |
| **psbNeU29SF** | Atha | F | F | **ed+ ^2^** | **ed+** | ed | **no** | F | **no** | **no** | **no** | **no** |
| **rbcLeU785AV** | Atha | V | V | ? | **no** | A | A | A | A | A | A | A |
| **rpl2eU2TM** | Atha | M | M | **ed+** | M | M | M | M | ed | ed | M | ed |
| **rpl20eU308SL** | Abel | L | ed | **no** | L | ed | L | ed | L | **no** | L | ed |
| **rpl23eU71SF** | Atha | F | **ed+** | ed | **ed+** | ed | F | **ed+** | F | ed | - | F |
| **rpl23eU89SL** | Atha | ed | **no** | ed | **no** | ed | ed | **ed+** | F | ? | - | F |
| **rpl32eU101PL** | Atha | L | L | L | **ed+** | L | L | L | L | L | L | L |
| **rpoAeU200SF** | Atha | ed | F | ed | ? | ? | ed | **ed+** | F | ed | ? | F |
| **rpoAeU836SL** | Atha | L | ed | ed | **ed+** | ? | ed | ed | L | ed | L | L |
| **rpoBeU29SF** | Paph | ? | ? | **no** | **no** | ? | **no** | **no** | ? | ed | ? | **no** |
| **rpoBeU566SL** | Atha | L | L | ed | **ed+** | ed | L | L | ed | ed | ed | ed |
| **rpoBeU623PL** | Atha | L | L | ed | L | L | L | L | **no** | ed | L | ed |
| **rpoBeU1831HY** | Paph | Y | Y | Y | Y | Y | Y | Y | **no** | Y | Y | **no** |
| **rpoBeU2432SL** | Atha | ed | ed | ed | ed | ed | L | L | L | ed | **ed+** | L |
| **rpoC1eU488SL** | Atha | ed | L | **ed+** | **ed+** | **ed+** | **ed+** | L | L | ed | **ed+** | L |
| **rps2eU134TI** | Abel | I | **ed+** | ed | I | ed | ed | ed | **ed+** | ed | ed | **ed+** |
| **rps2eU248SL** | Atha | L | ed | **ed+** | ed | ed | ed | ed | L | L | ed | L |
| **rps2eU266TI** | Atha | I | I | I | **no** | I | I | I | I | I | I | I |
| **rps2eU668SF** | Atha | F | F | **no** | F | V | F | F | F | **no** | F | F |
| **rps4eU463PS** | Atha | S | S | **no** | S | S | S | S | S | S | S | S |
| **rps7eU451LF** | Paph | F | F | F | F | F | F | F | **no** | F | F | **no** |
| **rps12eU221SL** | Atha | L | L | L | L | ed | L | L | L | **ed+** | L | L |
| **rps14eU194SL** | Abel | **no** | L | L | L | **no** | **no** | L | L | L | L | L |
| **rps18eU70PS** | Paph | S | S | S | S | S | S | S | **ed+** | S | S | **ed+** |
|  |  | **Atha** | **Abel** | **Cnuc** | **Csat** | **Ghir** | **Hbra** | **Ntab** | **Osat** | **Paph** | **Psat** | **Zmay** |
