# Supplementary material for: Plant organelle RNA editing and its specificity factors: enhancements of analyses and new database features in PREPACT 3.0
Source: BMC Bioinformatics. 2018 Jul 3;19:255. doi: 10.1186/s12859-018-2244-9 (PMC6029061; doi:10.1186/s12859-018-2244-9)

**Additional file 3.**

Top of the “Alignment prediction” output for the *atp9* query example in additional file 2. As in the BLASTX mode (Fig. 2), the output is similarly arranged into individual tabs for the chosen references plus a comparative commons tab, here selected for display. Links to editing predictions for the individual sequences are provided under the respective tabs. In the example case, editing predictions from the nine chosen references are fully congruent for 11 candidate editing sites in *Liriodendron tulipifera*.


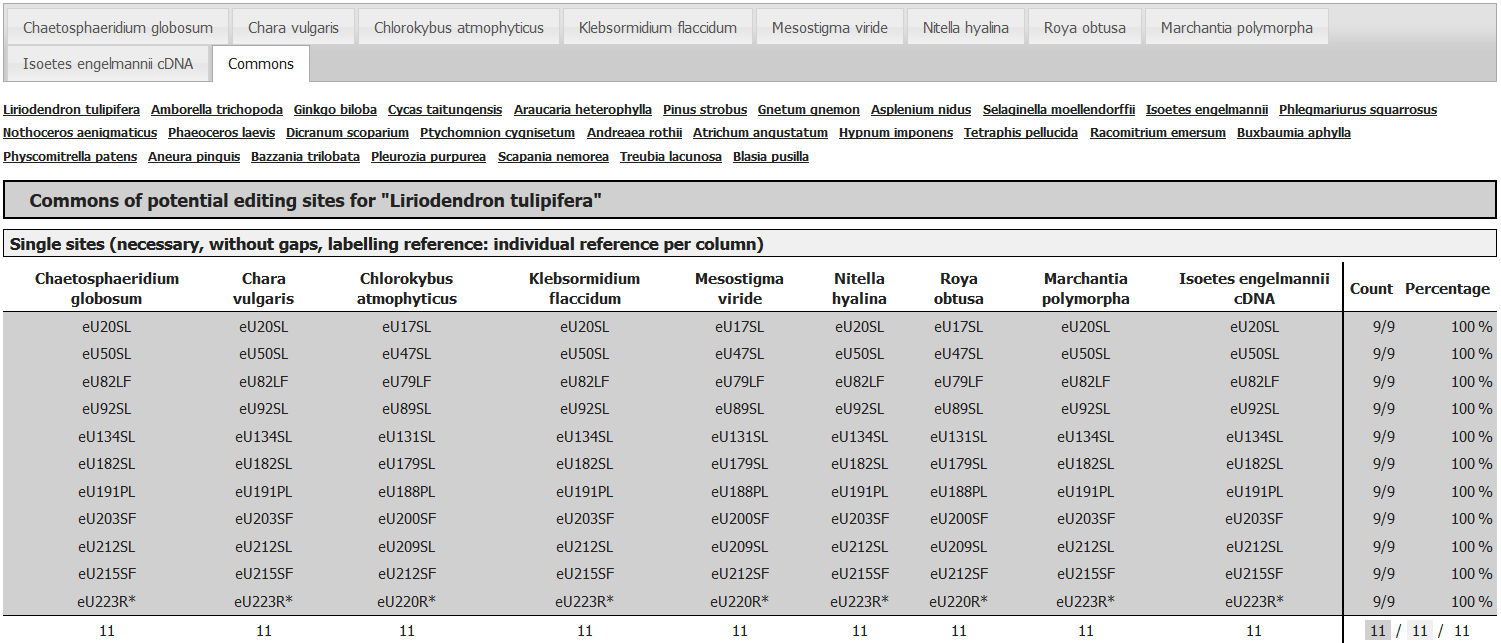

Supplement: Supplementary file 3 — Alignment prediction output. An example for the output of a multiple-query alignment for different references. (DOCX 79 kb) [file 12859_2018_2244_MOESM3_ESM.docx]
