# Supplementary material for: Plant organelle RNA editing and its specificity factors: enhancements of analyses and new database features in PREPACT 3.0
Source: BMC Bioinformatics. 2018 Jul 3;19:255. doi: 10.1186/s12859-018-2244-9 (PMC6029061; doi:10.1186/s12859-018-2244-9)

**Additional file 4.**

TargetScan output for querying positions in the immediate vicinity of editing sites, exemplarily shown for position -1 upstream of all angiosperm chloroplast RNA editing sites in the updated PREPACT database. Here, weighting is arbitrarily set to 100 for the editing site and 40, 30, 20 or 10 for A, C, G or T, respectively, at position -1. Shown is the output for ranks 256 to 265, including the only two editing sites preceded by a G in position -1 (hits 260 and 261 scoring 120). Altogether, there are 69 occurrences of A, 190 C, 2 G and 620 T in position -1 among the altogether 881 RNA edting sites of the 14 angiosperm chloroplast editome data set (Fig. 7A).


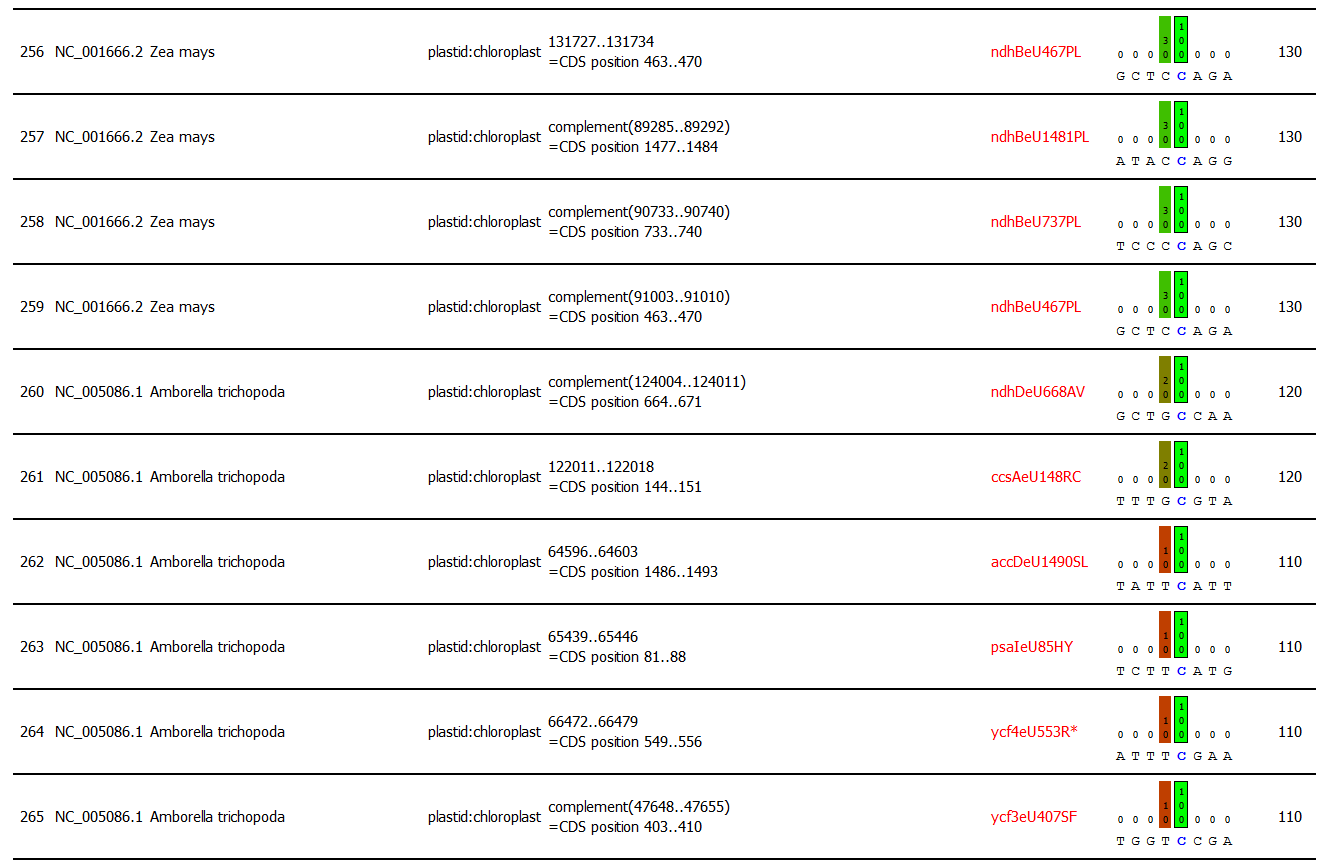

Supplement: Supplementary file 4 — TargetScan of Editing environment. An example illustrating the use of TargetScan to identify nucleotide bias in the immediate environment of editing sites in position -4 to +3 as discussed in the text. (DOCX 87 kb) [file 12859_2018_2244_MOESM4_ESM.docx]
